# Prolactin selectively transported to cerebrospinal fluid from blood under hypoxic/ischemic conditions

**DOI:** 10.1371/journal.pone.0198673

**Published:** 2018-06-27

**Authors:** Naoto Tani, Tomoya Ikeda, Miho Watanabe, Junko Toyomura, Akihiro Ohyama, Takaki Ishikawa

**Affiliations:** 1 Department of Legal Medicine, Osaka City University Medical School, Osaka, Japan; 2 Forensic Autopsy Section, Medico-legal Consultation and Postmortem Investigation Support Center, Osaka, Japan; 3 Department of NDU Life Sciences, School of Life Dentistry at Tokyo, The Nippon Dental University, Tokyo, Japan; 4 Field of Oral and Maxillofacial Surgery and System Medicine, Course of Clinical Science, Nippon Dental University, Graduate School of Life Dentistry at Niigata, Niigata, Japan; Massachusetts General Hospital/Harvard Medical School, UNITED STATES

## Abstract

**Aim:**

The aim of this study was to determine and to verify the correlation between the amount of prolactin (PRL) levels in the blood and in the cerebrospinal fluid (CSF) by various causes of death as an indicator for acute hypoxia in autopsy cases. It is to confirm the cause of the change in prolactin level in CSF by in vitro system.

**Materials and methods:**

In autopsy materials, the PRL levels in blood from the right heart ventricle and in the CSF were measured by chemiluminescent enzyme immunoassay, and changes in the percentage of PRL-positive cells in the pituitary gland were examined using an immunohistochemical method. Furthermore, an inverted culture method was used as an in vitro model of the blood-CSF barrier using epithelial cells of the human choroid plexus (HIBCPP cell line) and SDR-P-1D5 or MSH-P3 (PRL-secreting cell line derived from miniature swine hypophysis) under normoxic or hypoxic (5% oxygen) conditions, and as an index of cell activity, we used Vascular Endothelial Growth Factor (VEGF).

**Results and discussion:**

Serum PRL levels were not significantly different between hypoxia/ischemia cases and other causes of death. However, PRL levels in CSF were three times higher in cases of hypoxia/ischemia than in those of the other causes of death. In the cultured cell under the hypoxia condition, PRL and VEGF showed a high concentration at 10 min. We established a brain-CSF barrier model to clarify the mechanism of PRL transport to CSF from blood, the PRL concentrations from blood to CSF increased under hypoxic conditions from 5 min. These results suggested that PRL moves in CSF through choroidal epithelium from blood within a short time. PRL is hypothesized to protect the hypoxic/ischemic brain, and this may be because of the increased transportation of the choroid plexus epithelial cells.

## Introduction

Prolactin (PRL) is a hormone primarily secreted by lactotrophs in the anterior pituitary gland, with the exception of placental PRL [1]. PRL secretion is usually regulated by PRL suppressors such as dopamine from the hypothalamus [2–4]. PRL secretion increases, when dopamine production and transportation are impaired as a result of hypothalamic dysfunction resulting from drugs, hypoxic conditions, except in prolactinoma of the pituitary [5]. PRL has a wide variety of effects, for example, it is known that PRL participates in the immune response, controls osmotic pressure and vascularization, and promotes neurogenesis in maternal and fetal brains [6–9]. Furthermore, studies on hyperprolactinemia under conditions of hypoxemia have been reported [10–12]. It has been suggested that hypoxia strongly effects on PRL levels during death. However, in the endocrinological field, our literature search did not find any studies that comprehensively examined changes in the levels of PRL in the blood or in the CSF. We clarified the correlation between PRL levels and various cause of death, excluding drug use and prolactinoma, and verified the effectiveness of PRL as an indicator for hypoxic conditions.

Therefore, we examined PRL using several autopsy samples and a cell culture of the blood–CSF barrier model to clarify the significance of existence PRL in the blood and CSF under hypoxic conditions. We established hypoxic culture using pituitary cells, and a blood–CSF barrier model was prepared to investigate changes in blood and CSF PRL levels under hypoxic conditions.

We evaluated the cell activity under hypoxic conditions according to the experimental model. In our examination, VEGF was measured together with PRL, though several markers of cell activity are known [13]. VEGF is a group of glycoproteins involved in angiogenesis and is known to be elevated by the action of hypoxia-inducible factor 1 (HIF 1), when cells become hypoxic [14]. Therefore, VEGF is considered to be suitable for the evaluation of hypoxic cell activity [15]. Using these methods, we clarified the mechanism of change in the PRL concentration in blood and CSF under hypoxic condition in vivo and in vitro.

## Materials and methods

### Ethics statement

This study was evaluated by the Independent Ethics Committee of the Osaka City University Graduate School of Medicine. According to the Independent Ethics Committee of the Osaka City University Graduate School of Medicine, informed consent from opt-out for the autopsy data analysis was approved.

### Autopsy samples

Serial autopsy cases were examined within 48 h postmortem at our institution. There were 118 cases (90 males and 28 females), and the median age was 70 years (range, 0–100 years). The specimens were collected aseptically using syringes. Blood was collected from the right heart chamber and the cerebrospinal fluid (CSF). The blood samples were centrifuged immediately to separate the serum and then stored at −20°C until use [16, 17]. The causes of death were classified according to the findings of the complete autopsy and macromorphological, micropathological, and toxicological examinations as follows: organ damage due to blunt injury (n = 42; acute blunt injury, n = 7; subacute blunt injury, n = 27; prolonged blunt injury, n = 8), hemorrhage shock by sharp instrument injury (n = 13; acute sharp instrument injury, n = 5; subacute sharp instrument injury, n = 8), burns caused by fire (n = 30), hypoxia/ischemia caused by asphyxia (n = 19; hanging/strangulation, n = 10; others, n = 9), drowning (n = 7), and acute ischemic heart disease (n = 8). The case profiles are presented in Table 1. For each cause of death, clearly verifiable cases with well-established pathological evidence without any significant complications were included.

**Table 1:** Case profiles.

Cause of death		n	Male/ female	Age (years)		Survival time (h)	Postmortem time (h)		Serum (median)	CSF (median)
				Range	Median		Range	Median		
Blunt injury*	**Acute**	**7**	**7/0**	**20–84**	**63**	**0.5**	**6–48**	**24**	**8.48–63.1 (28.9)**	**3.16–74.2 (6.66)**
	**Subacute**	**27**	**17/10**	**1–100**	**71**	**1–24**	**6–72**	**24**	**0.498–75.5 (15.2)**	**1.71–53.0 (6.84)**
	**Prolonged**	**8**	**6/2**	**30–92**	**71**	**24–240**	**12–48**	**30**	**1.02–81.3 (3.91)**	**1.37–26.3 (4.28)**
**Sharp** **instrument injury**	**Acute**	**5**	**4/1**	**45–86**	**62**	**0.5**	**12–60**	**24**	**3.64–40 (18.8)**	**1.23–13.8 (6.08)**
	**Subacute**	**8**	**8/0**	**47–91**	**71.5**	**1–6**	**6–36**	**30**	**12.4–44.8 (19.6)**	**1.90–25.7 (17.6)**
**Burn**	**CO-Hb < 30%**	**6**	**5/1**	**44–87**	**71.5**	**0.5**	**12–36**	**18**	**8.62–44.8 (22.7)**	**1.78–16.7 (5.21)**
	**CO-Hb = 30–60%**	**12**	**8/4**	**50–88**	**72**	**0.5**	**12–36**	**24**	**7.52–162 (20.5)**	**2.23–35.0 (9.07)**
	**CO-Hb > 60%**	**12**	**9/3**	**61–91**	**76**	**0.5**	**12–36**	**24**	**9.23–88.7 (24.1)**	**2.49–35.6 (8.76)**
**Hypoxia/ischemia** **by Asphyxia**	**Hanging**	**5**	**3/2**	**37–70**	**51**	**0.5**	**12–60**	**48**	**1.84–97.4 (10.4)**	**8.20–45.1 (25.3)**
	**Strangulation**	**4**	**2/2**	**58–87**	**72**	**0.5**	**24–48**	**42**	**8.81–67.6 (17.8)**	**20.0–57.1 (27.0)**
	Others**	**9**	**6/3**	**6–86**	**67**	**0.5**	**6–48**	**36**	**9.11–73.2 (17.7)**	**7.50–38.4 (15.6)**
**Drowning**		**7**	**6/0**	**33–85**	**65**	**0.5**	**12–48**	**36**	**2.89–25.2 (10.8)**	**1.41–35.5 (5.6)**
**Acute cardiac death**		**8**	**8/0**	**0–75**	**45**	**0.5**	**12–36**	**24**	**5.04–59.8 (15.8)**	**3.13–24.8 (6.16)**
**Total**		**118**	**90/28**	**0–100**	**70**	**0.5**	**6–72**	**24**	**0.498–162 (17.6)**	**1.23–74.2 (8.34)**

* Blunt injury: head (n = 18) and non-head injury (n = 24)

### Biochemical analysis

Amount of PRL in serum and CSF were measured by chemiluminescent enzyme immunoassay using PATHFAST^®^ (LSI Medience Corporation, Tokyo, Japan) according to the manufacturer’s protocol. For these measurements, the clinical serum reference range was 4.98−26.4 ng/mL for males and 1.74−26.8 ng/mL for females. Amount of PRL in the conditioned medium of SDR-P-1D5 cells was measured by prolactin EIA Kit (A05101; Bertin Bioreagent, Montigny le Bretonneux, France), and in the conditioned medium of MSH-P3 cells was measured by pig prolactin ELISA KIT (MBS705496, MyBioSource, CA, USA). Amounts of vascular endothelial growth factor (VEGF) in the conditioned medium of SDR-P-1D5 cells was measured by rat VEGF ELISA KIT (ab100786; Abcam, Tokyo, Japan), and in the conditioned medium of MSH-P3 cells was measured by pig VEGF 164 ELISA KIT (ab218298; Abcam, Tokyo, Japan).

### Quantification of *mRNA* in the pituitary gland

Pituitary gland tissue specimens were immediately immersed in 1 mL of RNA stabilization solution (RNAlater^TM^, Ambion, Austin, TX, USA) and stored at −80°C until use. Total RNA was isolated using Isogen (Nippon Gene, Toyama, Japan) according to the manufacturer’s instructions. cDNA copies of total RNA were synthesized using the High-Capacity RNA-to-cDNA kit (Applied Biosystems, Foster City, CA, USA). The reaction mixture included 9 μL samples of total RNA, 10.0 μL 2× RT buffer, and 1.0 μL 20× RT enzyme mix. Conditions for reverse transcription were as follows: 37°C for 60 min and 95°C for 5 min [18]. A total of 20.0 μL of reaction mixture containing 10.0 μL TaqMan^®^ gene expression master mix (2×), 1.0 μL TaqMan^®^ gene expression assay (20×), 4 μL cDNA, and 5 μL H_2_O were added to each well of a Fast 96-well reaction plate (0.1 mL). Quantitative reverse transcription–polymerase chain reaction (PCR) was performed using primers for *PRL* (TaqMan assay ID: Hs00168730_m1) on a StepOnePlus real-time PCR system (Applied Biosystems). The threshold cycle (Ct) was calculated automatically by the instrument software with a threshold value of 0.2 [19]. The stability of endogenous reference genes in the pituitary gland was tested. For this study, the endogenous reference gene used was *SDHA* (TaqMan assay ID: Hs00188166_m1), which was the most stable of the endogenous reference genes analyzed (*Actinβ*, *B2M*, *GAPDH*, *HMBS*, *HPRT1*, *PPIA*, *SDHA*, and *TBP*). TaqMan^®^ Gene Expression Assays were purchased from Applied Biosystems and tested according to the manufacturer’s protocol (S1 Table).

### Toxicological analyses

Blood CO-Hb saturation (%) was analyzed using a CO-oximeter system (ABL80FLEX system; Radiometer Corp., Tokyo). Blood cyanide and alcohol levels were determined using headspace gas chromatography/mass spectrometry [20, 21]. Drug analyses were performed using gas chromatography/mass spectrometry [22, 23].

### Immunohistochemistry

Serial 4-μm sections were prepared from formalin-fixed, paraffin-embedded tissue specimens obtained from the pituitary and choroid plexus. Rabbit polyclonal antibody to PRL (ab64377; Abcam) was used at empirically determined dilutions [24]. Immunoreactivity was achieved by the polymer method using Dako Envison+ Dual Link System-HRP (K4063; Dako, California, USA) and Dako Liquid DAB+ Substrate Chromogen System (K3468; Dako) according to the manufacturer’s instructions. The percent positivity in the pituitary was estimated as the percentage of positive PRL cells, which was calculated as follows: number of positive PRL cells / total number of anterior pituitary cells × 100 [25].

### Cell culture system and PRL measurements

SDR-P-1D5 cell line was established from the pituitary of GH-deficient spontaneous dwarf rats (RCB3600) [26] and MSH-P3 cell line was established from the pituitary glands of 90-day-old fetuses of miniature swine were used in this experiment. Regarding the establishment of MSH-P3 cells, the pituitary glands of 90-days-old fetuses of miniature swine were removed, cut into small pieces with surgical blades, and dissociated with 0.1% trypsin-0.02% ethylenediaminetetraacetic acid/PBS (-) at 37°C for 30 min. After adding a little fetal bovine serum (FBS), fragments were further isolated into single cells by vigorous pipetting. The dissociated cells and small fragments were centrifuged at 340 × g for 5 min at room temperature. The precipitate was resuspended in growth medium (GM) and cultured in 60 mm dishes in a CO_2_ incubator (4.7% CO_2_, 95.3% air) at 37°C. The GM used for the SDR-P-1D5 or MSH-P3 cell line consisted of DMEM/F12 supplemented with 15% FBS (Sigma, Lot no. 12E261, 100 μM of GlutaMAX^TM^, 0.1% Modified Eagle Medium Non-Essential Amino Acids, 50 U/mL penicillin, 50 μg/mL streptomycin, and 0.25 μg/mL Fungizone (all purchased from Thermo Fisher Scientific Inc.). The number of fibroblastic cells gradually decreased over the course of the culture, resulting in the establishment of the MSH-P3 cell line. After the cells reached confluency, they were split and incubated at a ratio of 1:3 into fresh GM. The GM was changed twice a week. The experimental medium consisted of DMEM/F12 supplemented with 5% albumin, without an antibacterial agent. Cells were cultured in a humidified atmosphere of 4.7% CO_2_ and 5% O_2_ at 37°C. The cell count was measured using a Burker-Turk counting chamber following a dye exclusion test and was adjusted to 1.4 × 10^6^ cells/mL until 90 min of culture. Perfusion culture was performed using a circumfusion apparatus for the measurement of hormone secretion within a short period of time. Culture fluid PRL and vascular endothelial growth factor (VEGF) levels were subsequently measured using ELISA.

### Choroid plexus epithelial cell-based model

**Fig 1:**
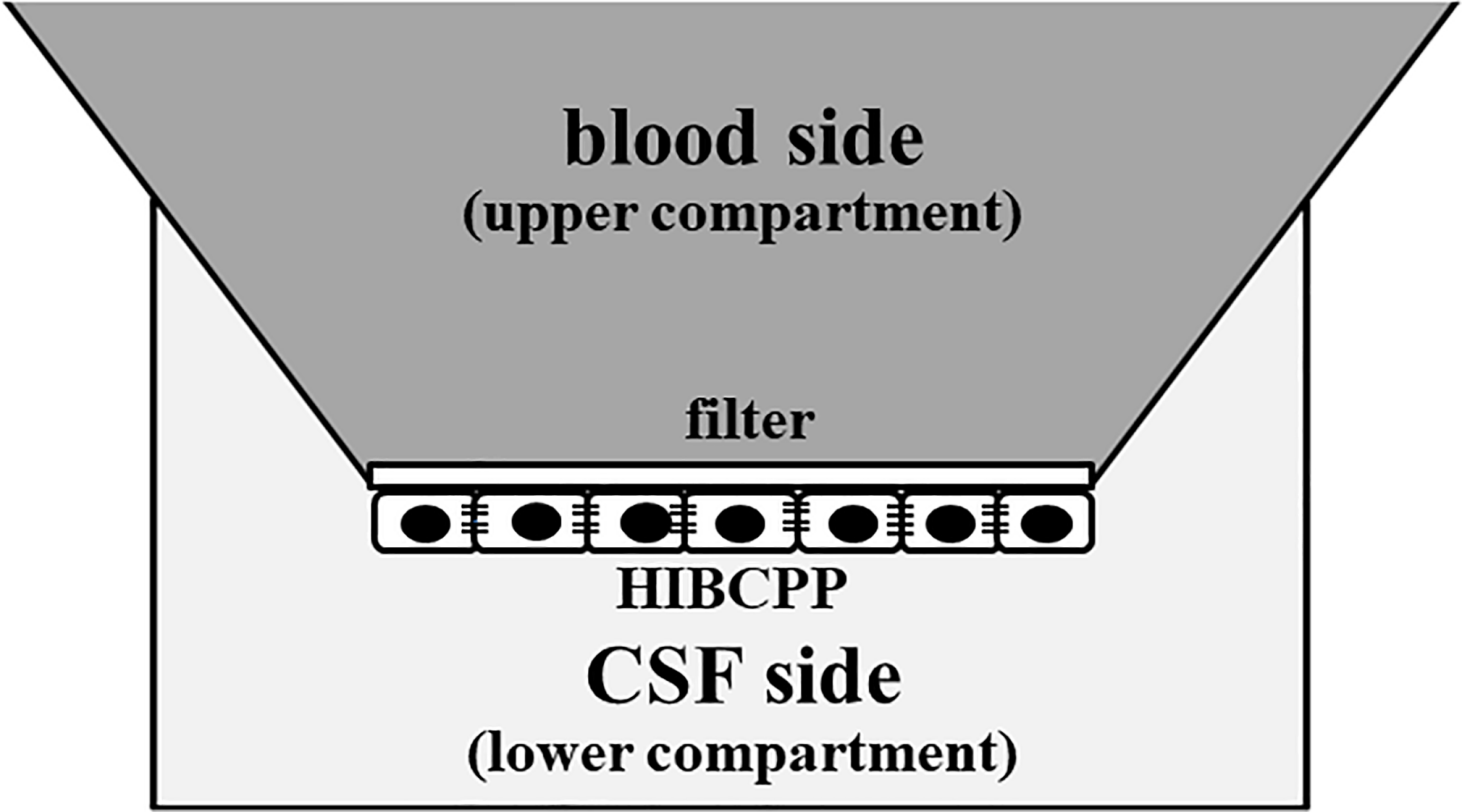
Schematic view of the choroid plexus epithelial cell culture method as a human blood cerebrospinal fluid barrier model.

The human malignant choroid plexus papilloma cell line (HIBCPP) was used as a choroid plexus epithelial cell-based model of the human blood CSF barrier (BCB). HIBCPP were cultured in DMEM F12 supplemented with 4 mM L-glutamine, 100 U/mL penicillin and 100 μg/mL streptomycin, 15% heat-inactivated fetal calf serum (FCS) [HIBCPP-medium with 15% FCS]. Because HIBCPP cells have been reported to change doubling time with increasing passages [27], only cells between passages 33 and 37 were used. For the BCB model, the amounts of cells indicated in the respective experiments were seeded onto transwell filters (pore diameter 3.0 μm, pore density 2.0 × 10^6^ pores per cm^2^, membrane diameter 0.33 cm^2^; Greiner Bio-One, Frickenhausen, Germany). For the transwell filter system, cells were seeded onto the lower filter well [28]. Because HIBCPP cells can form a monolayer, trypsinization and seeding of HIBCPP were extensively optimized to allow formation of a maximal proportion of a monolayer on the transwell filters (Fig 1). HIBCPP cells produce monolayers by forming connections between cells in the form of tight junctions [29]. Subsequently, cells were washed once every 2 days. Medium was added to the lower well not before day two after seeding. PRL of 100 ng/ml was dropped into the upper compartment, and the quantity of PRL in the lower compartment was measured from 5 to 30 min under conditions of normoxia and hypoxia (5% O_2_). Culture fluid PRL levels were measured using ELISA. Because these cells form tight junctions, PRL does not leak from the vacant space between the cells [29].

### Statistical analysis

Spearman’s rank correlation coefficient was used to compare two values, including PRL levels, age of the subjects, and postmortem time. For comparisons between groups, we used the nonparametric Mann−Whitney U test. Games−Howell test was used for analyses involving multiple comparisons. In this test, the line in each box represents the median, and the lines outside each box represent the 90% confidence interval. The maximum PRL levels in serum and CSF were log-transformed for graphical presentation only. Diagnostic relevance was estimated according to the values obtained for sensitivity, specificity, and accuracy (proportion of subjects correctly predicted). Youden’s index (sensitivity + specificity -1) was used to determine the best cut-off value. We evaluated the usefulness of PRL levels in the serum and CSF for differentiating between death due to hypoxia/ischemia by asphyxia and other causes of death, using receiver operating characteristic curves and the respective areas under the curve. The results are presented as medians. All analyses were performed using the SPSS 9.0 statistical package (SPSS Inc., Chicago, IL, USA). A *p*-value <0.05 was considered significant.

## Results

### Relationship between the PRL level and collection site

A slight correlation was observed between serum and CSF PRL levels (*r* = 0.221, *p* < 0.05). The PRL level was significantly higher in the serum (median, 17.6 ng/mL; range, 0.4−162.0 ng/mL) than in the CSF (median, 8.3 ng/mL; range, 1.2−74.2 ng/mL) (*p* < 0.0001) (S1 Fig).

### Relationship of PRL levels with sex, age, survival period, and postmortem period

PRL levels were lower in male patients than in female patients in both serum (male: median, 14.2 ng/mL; range, 0.4−139.0 ng/mL; female: median, 26.0 ng/mL; range, 1.5−162.0 ng/mL) and CSF (male: median, 8.1 ng/mL; range, 1.2−74.2 ng/mL; female: median, 12.2 ng/mL; range, 2.4−45.1 ng/mL) (*p* < 0.01, respectively). A slightly correlation was observed between the postmortem period and PRL levels of the serum (*r* = -0.228, *p* < 0.05) and CSF (*r* = 0.211, *p* < 0.05). No relationship was found between PRL levels and age or survival period in either the serum or CSF, respectively, except for serum PRL levels and survival period (r = -0.201, p < 0.05) (S1 Fig).

### Relationship between PRL levels and causes of death

**Fig 2:**
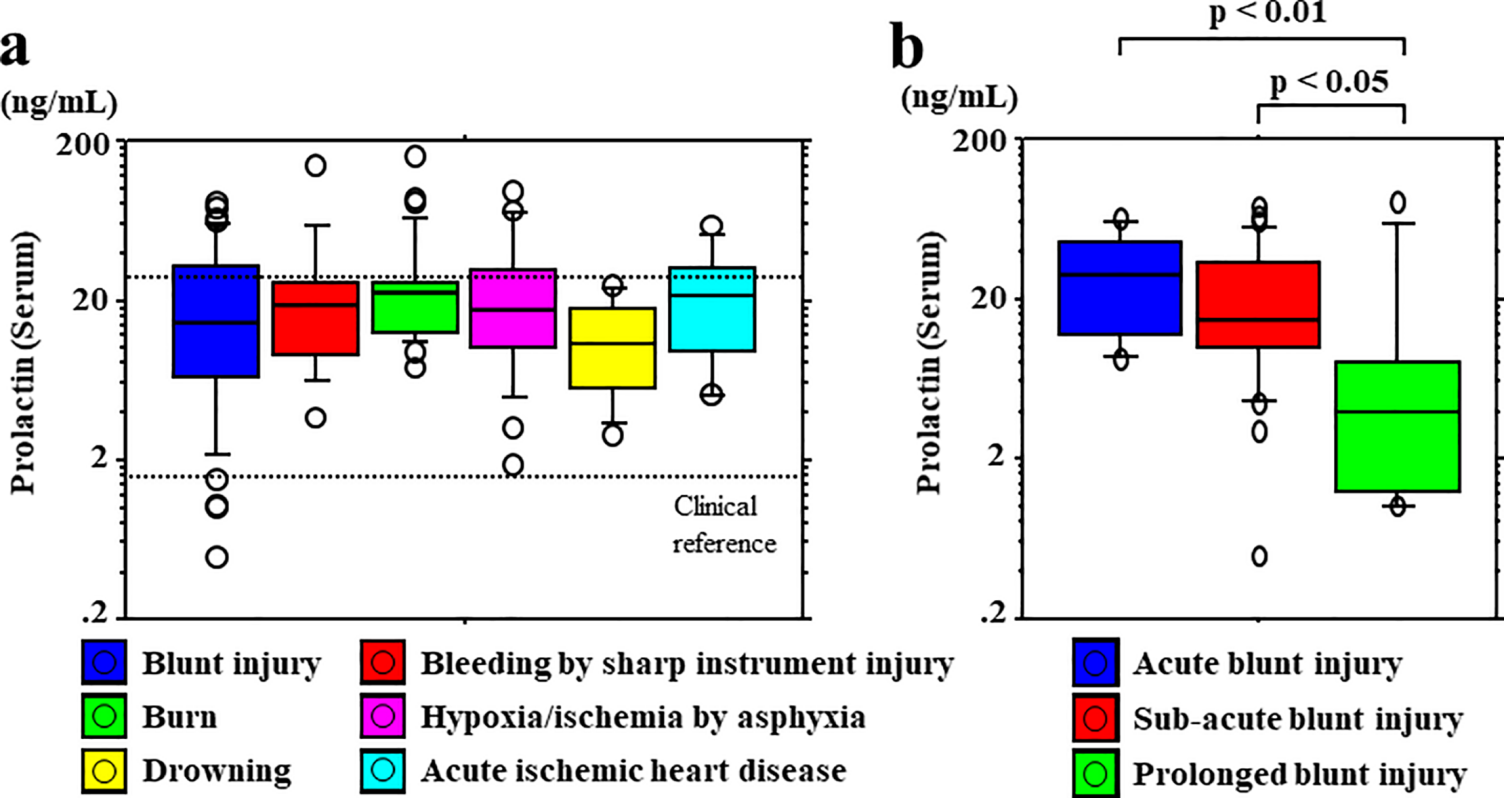
Postmortem serum PRL levels with regard to the causes of death. (a) No significant differences in serum prolactin (PRL) levels were observed according to the causes of death. (b) Serum PRL levels decreased according to the survival period in blunt injury cases, including those involving head and non-head injuries.

**Fig 3:**
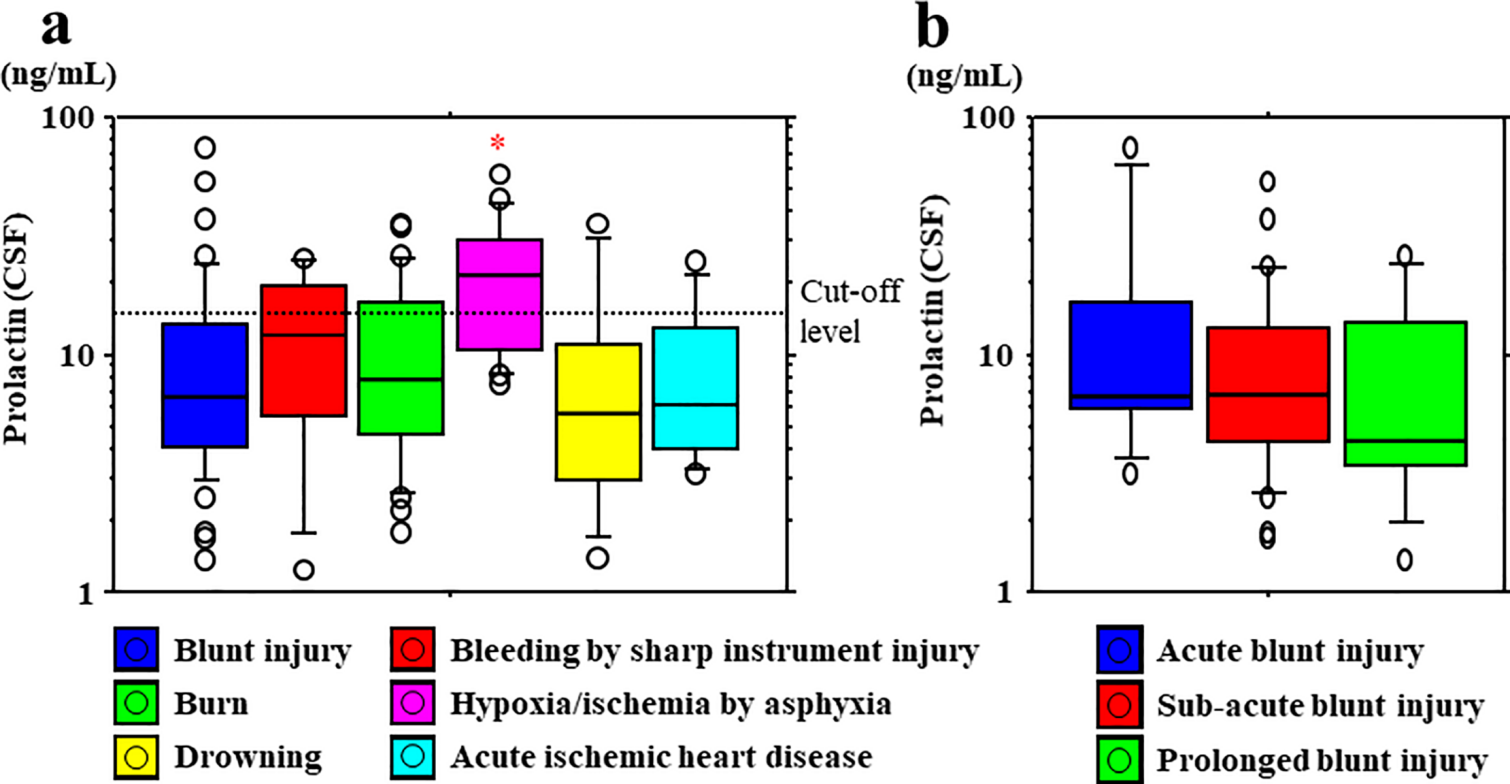
Postmortem CSF PRL levels with regard to the causes of death. (a) Cerebrospinal fluid (CSF) PRL levels are significantly higher in asphyxia cases than in cases involving other mechanisms of death. *Significantly higher: asphyxia versus other causes of death using the Mann−Whitney U test and Games−Howell test (*p* < 0.001 to *p* < 0.01). (b) No change was observed in CSF PRL levels according to the survival period in blunt injury cases, including those involving head and non-head injuries.

No significant differences in serum PRL levels and the causes of death were observed (Fig 2A). Serum PRL levels were not significantly different between cases of asphyxia-induced hypoxia/ischemia (median, 17.6 ng/mL; range, 1.8−97.4 ng/mL) and other mechanisms of death (median, 17.1 ng/mL; range, 0.4−162.0 ng/mL). However, serum PRL levels decreased according to the survival period in blunt injury cases, including those involving head and non-head injuries (Fig 2B). CSF PRL levels were higher in cases of asphyxia-induced hypoxia/ischemia (median, 21.8 ng/mL; range, 7.5−57.1 ng/mL) than in cases involving other mechanisms of death (median, 7.1 ng/mL; range, 1.2−74.2 ng/mL) (*p* < 0.05 to *p* < 0.001) (Fig 3A). A cut-off value of 15.4 ng/mL was identified to distinguish higher and lower PRL levels (asphyxia vs. other groups) for CSF samples (sensitivity, 0.68; specificity, 0.76) (Fig 3A). However, CSF PRL levels did not show any decrease according to the survival period in blunt injury cases, including those involving head and non-head injuries (Fig 3B).

### Relative quantification of *PRL* mRNA levels in the pituitary gland with regard to the causes of death

**Fig 4:**
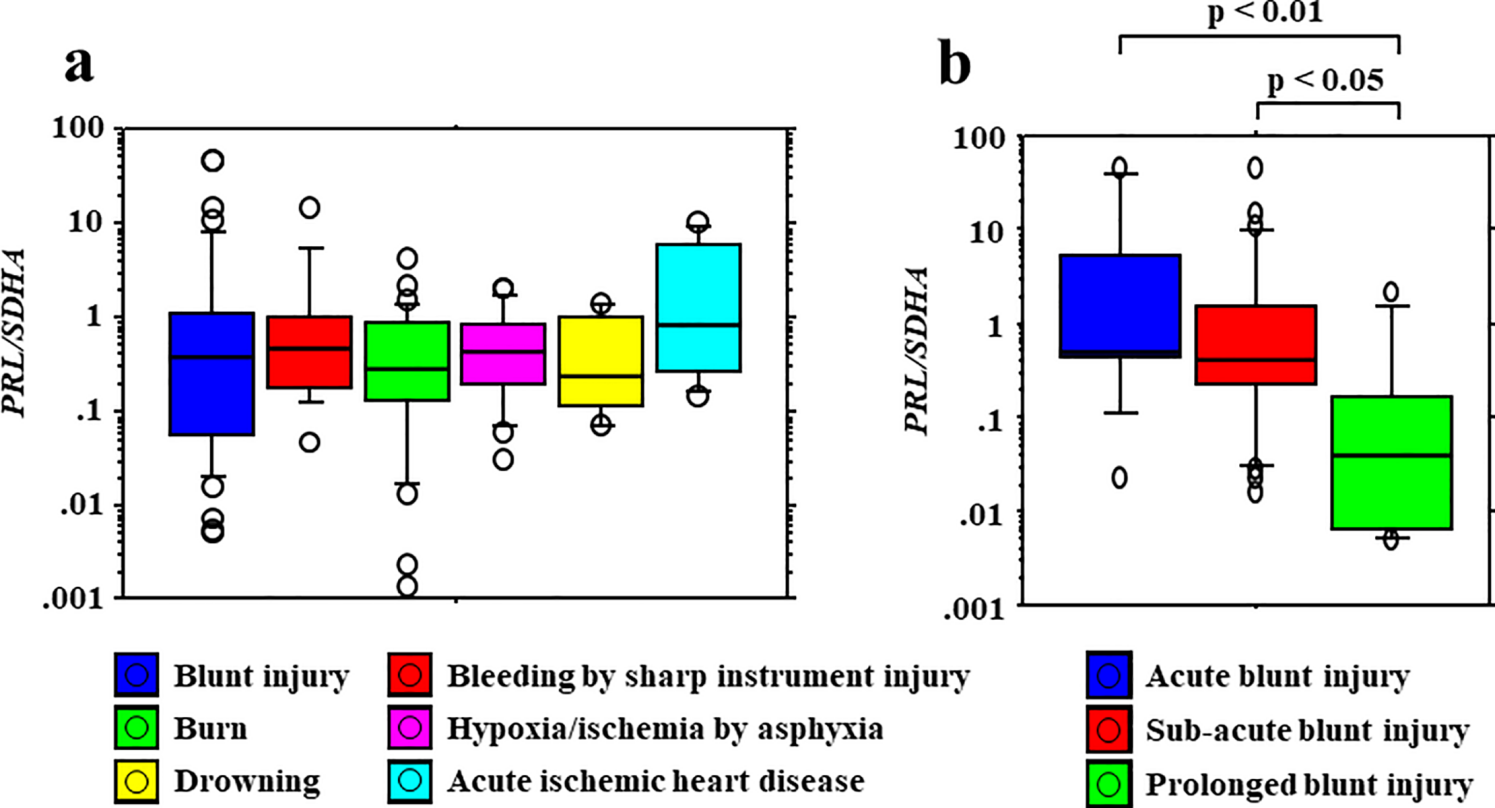
Relative quantification of *PRL* mRNA levels in the pituitary gland with regard to the causes of death. (a) No significant differences in *PRL* expression are observed among the causes of death. (b) *PRL* expression decreases according to the survival period in blunt injury cases, including those involving head and non-head injuries.

*PRL* expression was not associated with CSF PRL levels, although *PRL* expression was associated with serum PRL levels. There was no significant relationship between *PRL* expression and the causes of death (Fig 4A). However, *PRL* expression decreased according to the survival period in blunt injury cases including those involving head and non-head injuries (Fig 4B). These findings showed a tendency similar to those for serum PRL levels (Figs 2B and 4B).

### Immunohistochemical PRL-positivity ratio

There was no significant difference in the PRL-positivity ratio with regard to the causes of death (blunt injury [median, 25%; range, 4−51%], hemorrhagic shock by sharp instrument injury [median, 25%; range, 7−50%], burn caused by fire [median, 27%; range, 5−55%], hypoxia/ischemia caused by asphyxia [median, 23%; range, 3−32%], drowning [median, 18%; range, 11−30%], and acute ischemic heart disease [median, 17%; range, 5−31%] (S2 Fig).

### Amounts of PRL or VEGF in the conditioned medium of the SDR-P-1D5, or MSH-P3 cultured under the hypoxic conditions

**Fig 5:**
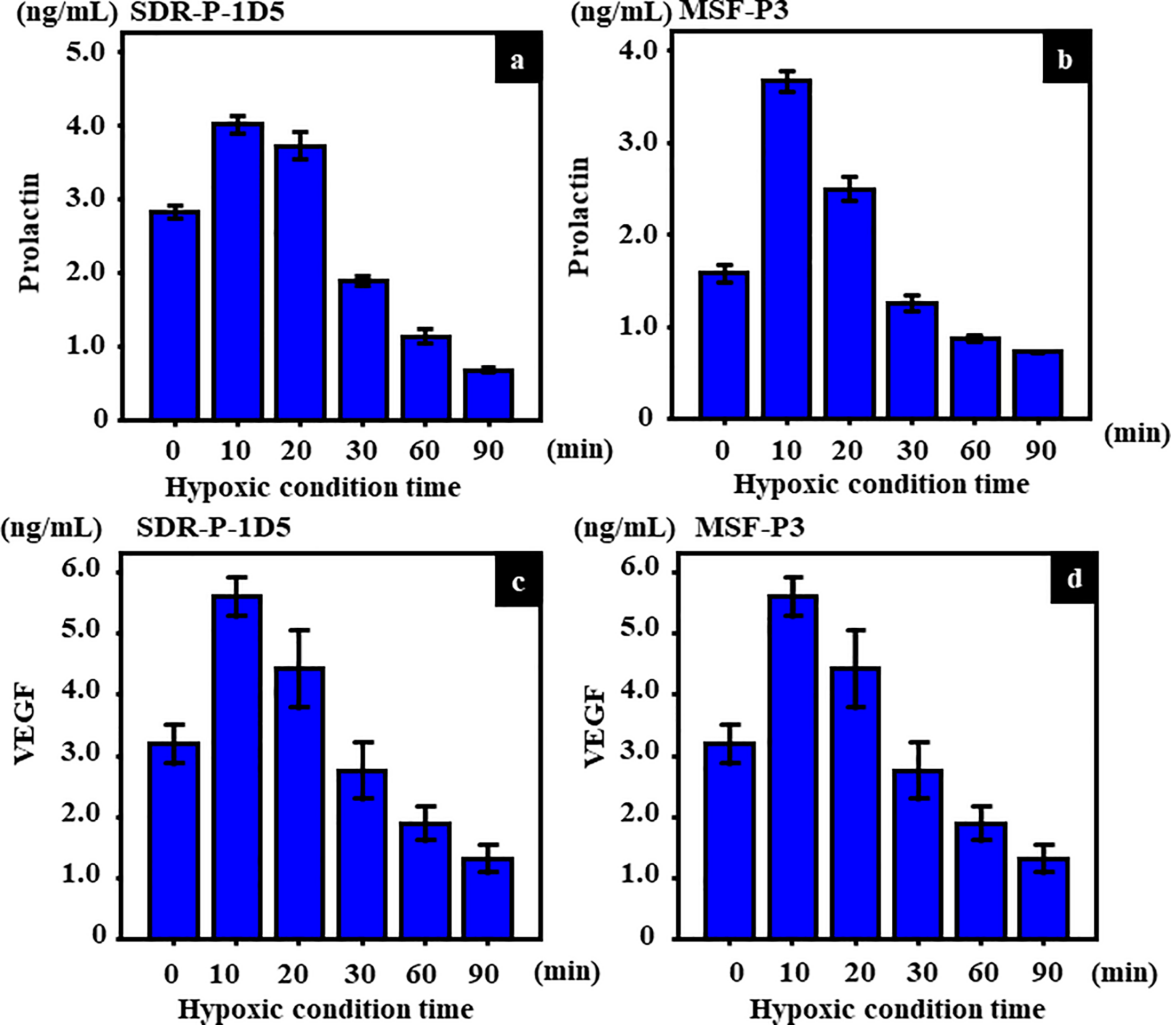
Amounts of PRL and VEGF in conditioned medium of SDR-P-1D5 and MSH-P3 cultured cells under hypoxic conditions. PRL levels under hypoxic conditions in (a) SDR-P-1D5 and (b) MSH-P3 cells. PRL were found to be at high levels in SDR-P-1D5 and MSH-P3 cells after 10 min incubation under 3% hypoxic conditions. However, PRL levels decreased after 20 min under hypoxic conditions. Measurement of vascular endothelial growth factor (VEGF) to confirm cell activity under hypoxic conditions in (c) SDR-P-1D5 and (d) MSH-P3 cells. Cell activity decreases following incubation for more than 20 min under hypoxic conditions. These experiments were performed five times.

**Fig 6:**
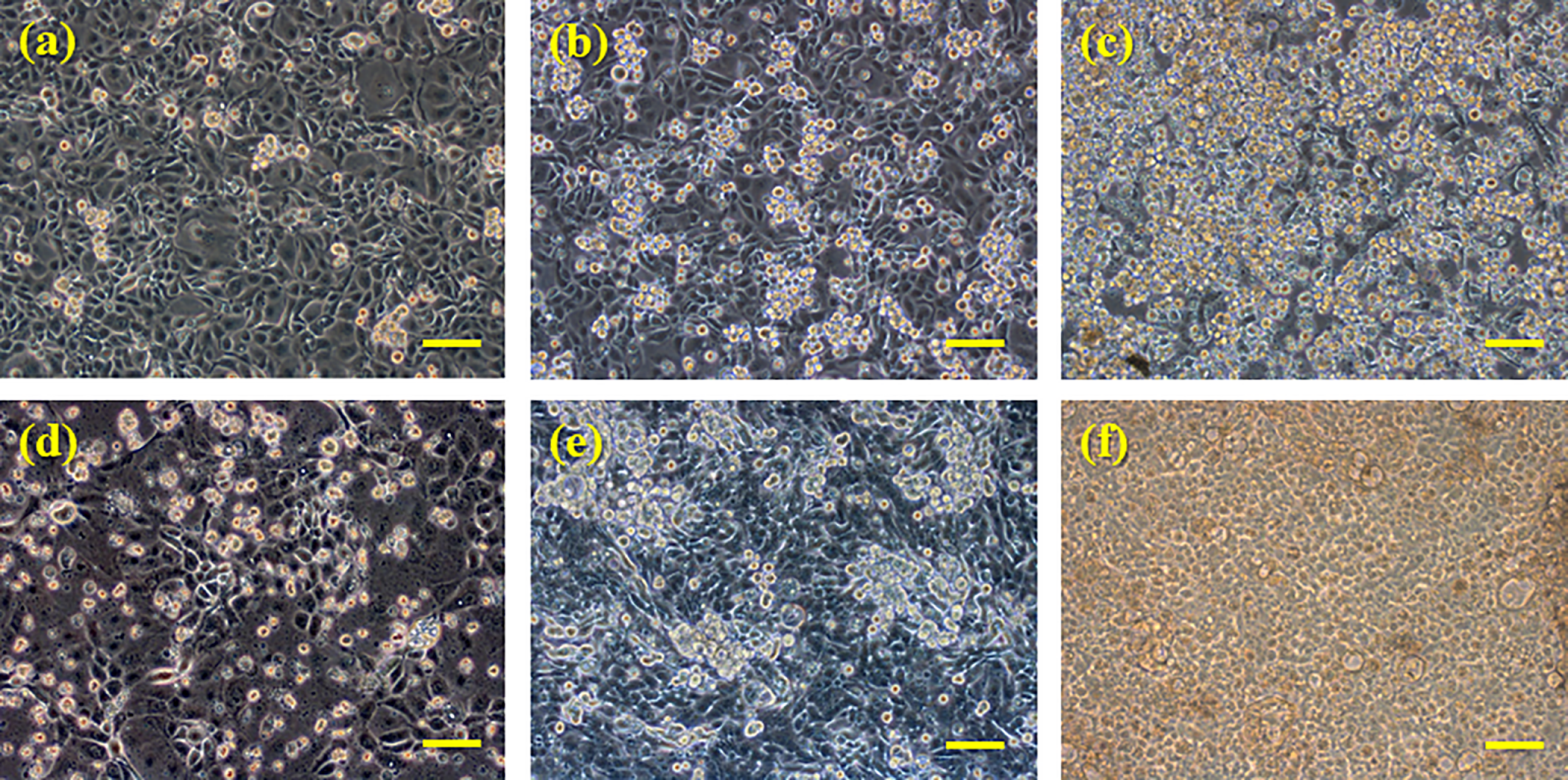
SDR-P-1D5 cells under phase-contrast microscopy. In SDR-P-1D5 cells, the number of the spherical cells was increased under hypoxic condition (b, c) compared with normoxia condition (a). A number of the spherical cells under hypoxic conditions were increased after 30 min (c) than 10 min (b). Similarly, in MSH-P3 cells, the number of the spherical cells was increased under hypoxic condition compared with normoxia condition (d). A number of the spherical cells under hypoxic conditions were increased after 30 min (f) than 10 min (e). Bar = 100 μm.

We cultured SDR-P-1D5 (rat pituitary) or MSH-P3 (swine pituitary) cells under hypoxic conditions (5% oxygen) for 90 min. Both SDR-P-1D5 and MSH-P3 cells showed high levels of PRL and VEGF following incubation for 10 min under hypoxic conditions (Fig 5A–5D). However, the PRL and VEGF levels gradually decreased after 20 min in both SDR-P-1D5 and MSH-P3 cells (Fig 5A–5D). With regard to the microscopy findings, the spherical cells increased at 30 min under hypoxic conditions compared with conditions of normoxia and 10 min of hypoxia both SDR-P-1D5 and MSH-P3, respectively (Fig 6A–6F). The spherical cells were alive because they could be cultivated again.

### PRL is transported to CSF from blood under hypoxic conditions

**Fig 7:**
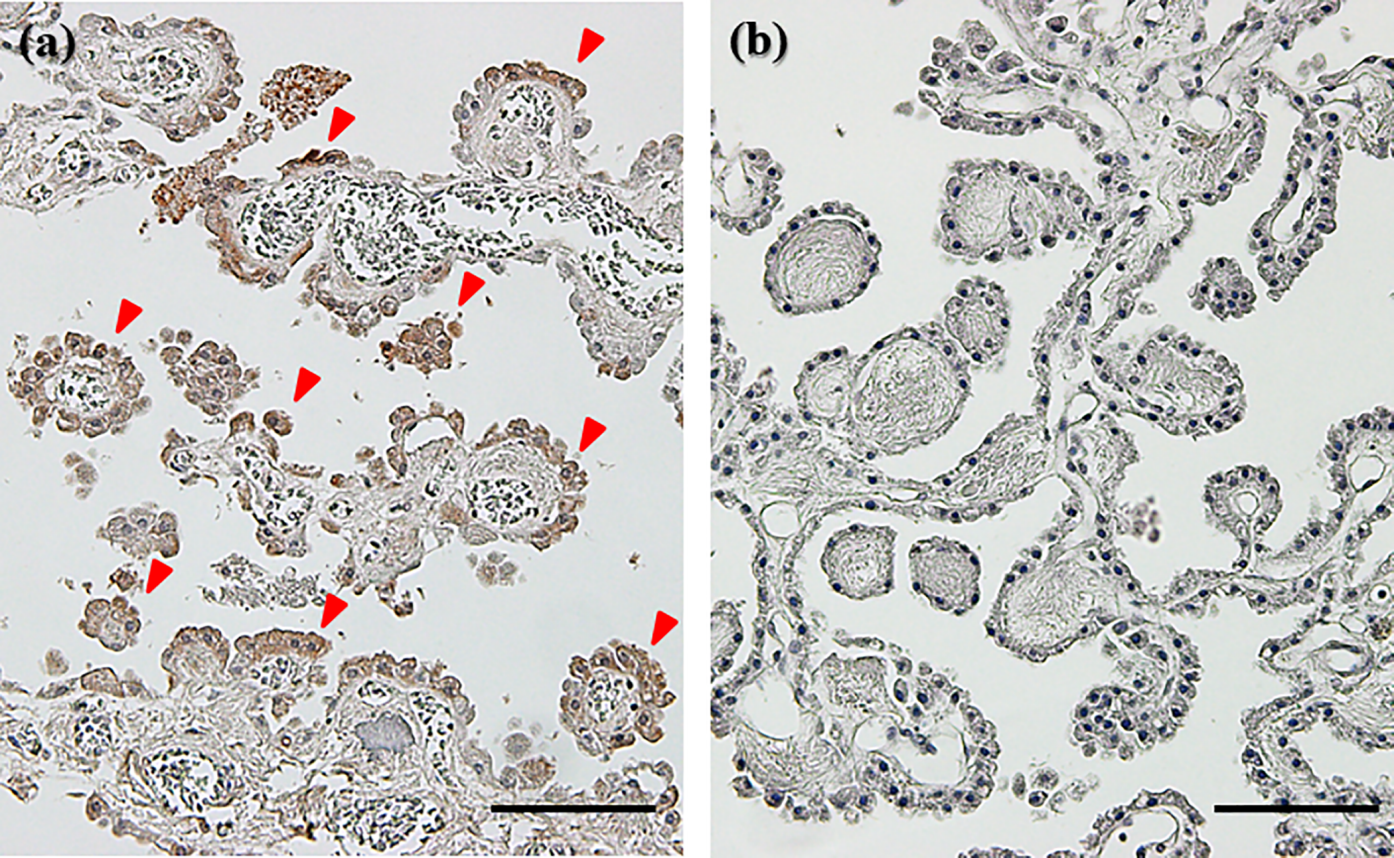
Immunostaining of PRL in the choroid plexus. Macrograph showing immunostaining for PRL in the choroid plexus in (a) cases of asphyxia-induced hypoxia/ischemia and (b) burn (CO-Hb < 30%) cases (original magnification × 100). Red arrowhead shows PRL-positive cells by DAB staining. Bar = 100 μm.

**Fig 8:**
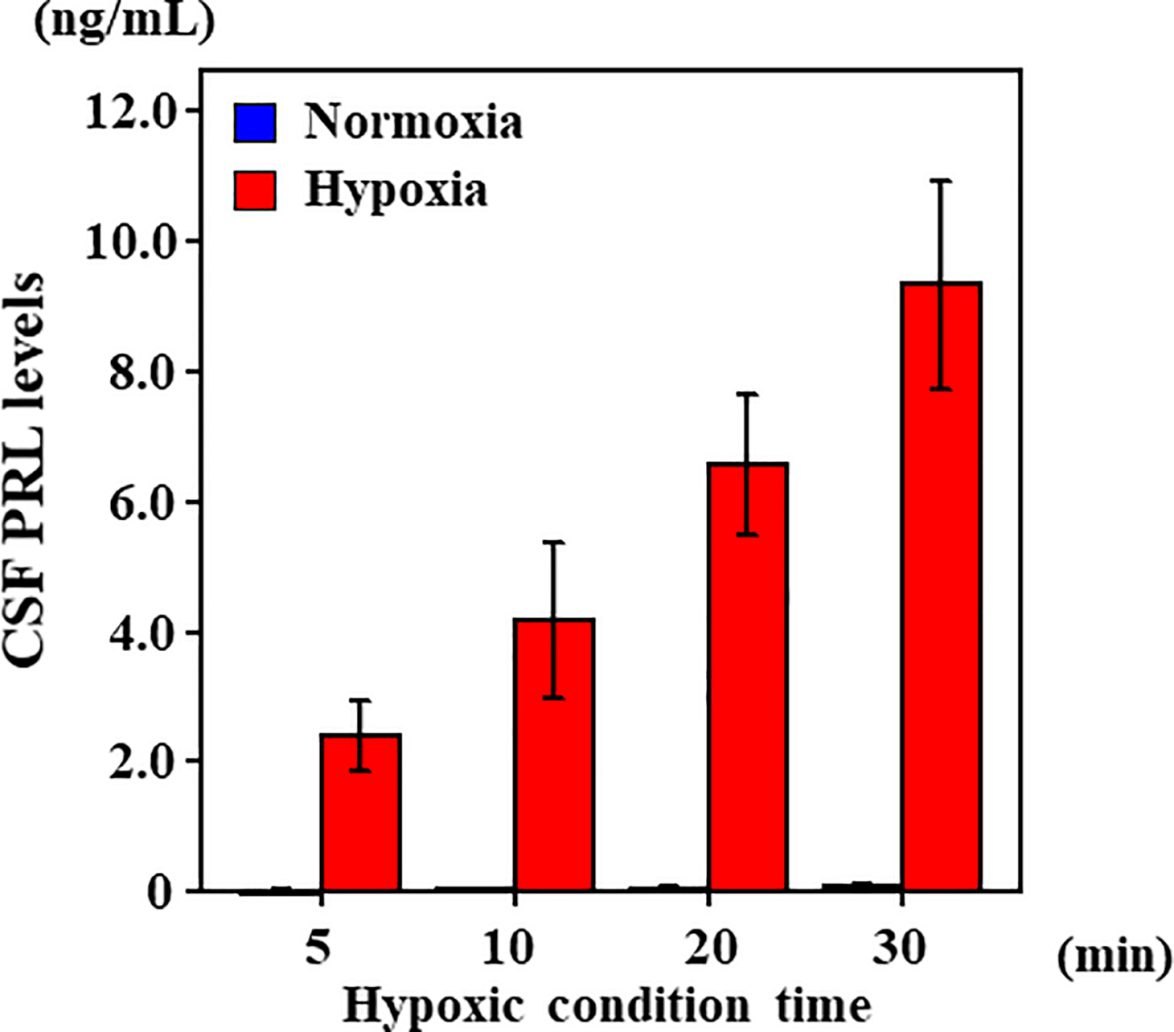
CSF PRL levels in a choroid plexus epithelial cell culture method as a human BCB model. BCB model experiments revealed no differences in PRL concentrations under normoxic conditions between 5 and 30 min in the lower compartment on the CSF side. On the other hand, PRL concentrations in hypoxic conditions increased from 5 to 30 min in the lower compartment (representing the CSF side). These experiments were performed six times.

Choroid plexus from cases of asphyxia-induced hypoxia/ischemia was found to be immunocyte chemical staining positive for PRL (Fig 7A), but immunocytochemical staining negative for other causes of death (Fig 7B). The results from the BCB model experiments revealed no differences in PRL concentrations under normoxic conditions between 5 and 30 min in the lower compartment on the CSF side. On the other hand, PRL concentrations in hypoxic conditions increased from 5 to 30 min in the lower compartment (representing the CSF side) (Fig 8).

## Discussion

No significant differences were observed in serum PRL levels and *PRL* mRNA levels among the different causes of death. However, PRL levels in serum decreased according to the survival period. Furthermore, there was no significant difference in PRL-positive cell ratio as determined by immunocytochemical staining among the causes of death. On the other hand, PRL levels in CSF were higher in cases of hypoxia/ischemia caused by asphyxia than in cases involving other causes of death.

PRL is secreted in the hypophyseal portal system by the anterior pituitary gland, and the half-life of the PRL is approximately 50 min. CSF PRL levels have been reported to correlate with blood PRL levels [30]. However, in our study, CSF PRL concentrations in cases of hypoxia/ischemia caused by asphyxia were higher than in other causes of death, although no differences in serum PRL levels and mRNA *PRL* levels among the various causes of death were observed.

In a hypoxic environment, PRL level in the blood and *PRL* mRNA level in the pituitary gland were not increased, however amount of PRL increased in CSF under hypoxia. These results suggested that PRL cells do not have the ability to synthesize PRL under hypoxic conditions. Instead, PRL stored in pituitary gland cells were released, PRL level in the CSF is thought to be increased by it passing through the BCB by hypoxic stimulation. In our previous study, we thought that the transportation of hormones (such as adrenocorticotropic, thyroid stimulating, or growth hormone) from blood to the CSF under hypoxic conditions is not increased. Only a prolactin is increased in CSF under hypoxia condition [31–33]. The reason for the increase in CSF PRL levels might be due to selective transport from blood to the CSF via the BCB under hypoxic conditions. PRL transport into the CSF has been reported to be independent of the PRL channel in the choroid plexus [28, 34, 35]. The results of the immunostaining for PRL in the choroid plexus were positive in asphyxia-induced hypoxia/ischemia cases. PRL in the CSF might protect the brain from hypoxic stress [36]. On the other hand, in blunt injury cases, including those involving head and non-head injuries, the levels of serum PRL and mRNA expression decreased in cases with long survival periods. CSF PRL levels tended to decrease with survival period, but there was no statistically significant difference. These results suggested that hypoxic conditions affect the hypothalamus–pituitary gland functional system, and/or BCB system, and PRL production and PRL channel function are controlled to suppression [37]. Although CSF PRL specifically increased during hypoxia/ischemia due to asphyxia, the pathophysiology may be different between hypoxia/ischemia due to asphyxia and circulatory failure as the last condition of the other causes of death. Almost other cause of death occurred were due to circulatory failure including hypoxia/ischemia, as the final pathophysiological condition. However, our results suggested that the cause of hypoxia/ischemia by asphyxia compared with heart failure as the final condition is greatly related to the change in the CSF PRL concentration. Hemorrhagic shock, heart disease, and drowning are known as conditions similar to those of hypoxia/ischemia in a patient. However, in death due to asphyxia by neck compression, the interception of the blood vessel and airway causes a severe systemic hypoxia/ischemic condition. In comparison, drowning leads to death from respiratory distress caused by alveolar damage [38]. Various heart diseases including ischemic heart disease are thought to cause death from hemodynamic distribution due to pump failure [39]. The main pathophysiology of the hemorrhagic shock is hypovolemia and vasoconstriction in the kidney, liver, intestinal tract and skeletal muscle. As a result, hypoperfusion and lack of oxygen supply at the cell level occur resulting in multiple organ failure [40]. The possibility that our results, such as asphyxiation, are caused by a systemic hypoxia/ischemia condition is supposed when we consider these pathologic differences.

In the present pituitary cell culture studies, culture cells were always exposed to new culture fluid under the circumfusion culture system, and hormone secretion from cultured cells does not influence the half-life or autocrine action of PRL [41]. With this method, we were able to determine the quantity of secretion at every point. As far as we know, human prolactin secreting cell line doesn’t exist. So we made sure of influence of hypoxia on prolactin secretion using rat prolactin secreting cell line. However, there was a possibility which is a peculiar reaction in rat, so swine prolactin secreting cell was also used and confirmed. An increase in PRL levels was noted 10 min after hypoxic exposure, whereas a decrease in secretion in cultured cells was observed 20 min after hypoxic exposure, suggesting that PRL can be secreted within a short period of time and that secretion is activated by hypoxic stress. Moreover, similar to PRL secretion, an increase in VEGF levels occurred 10 min after hypoxic exposure, with a decrease in secretion in cultured cells 20 min after hypoxic exposure. We evaluated VEGF as a cell function marker under hypoxia, it is suggested that adenohypophysial cell dysfunction due to hypoxia [42]. Several reports have described that hypoxia induces VEGF secretion in vitro [43–45]. It was believed that the decrease in PRL secretion that occurred 20 min after hypoxic exposure was associated with a decrease in cell function, as VEGF also decreased 20 min after hypoxic exposure. PRL secretion within a short period of time under hypoxic conditions, and it is considered that PRL secretion decreases because of a pituitary cell disorder under hypoxic conditions, as reflected by the morphology of cells. These results indicated that PRL stored in pituitary gland cells is released, and PRL level may increase in the CSF by it passing through the BCB upon hypoxic stimulation. The results of the choroid plexus epithelial cell-based (blood−CSF barrier) model experiments revealed no differences in PRL concentrations under conditions of normoxia between 5 and 30 min in the lower compartment (the CSF side) indicating that PRL did not transfer from the upper compartment to the lower compartment under normoxia conditions. On the other hand, PRL concentrations increased between 5 and 30 min under hypoxic conditions, and PRL transferred from the upper compartment to the lower compartment, suggesting therefore that PRL is selectively transported to the CSF from the blood under conditions of hypoxia. From our previous studies, we regard this transportation as being a PRL-specific phenomenon. It is known that brain edema generally occurs when the brain falls into a hypoxic condition [46]. At this point, PRL may transport to CSF from blood, adjust osmotic pressure to the brain, and act on neuroprotection [47].

## Conclusions

In conclusion, our observations form the postmortem PRL levels and cultured cell studies suggest that PRL is transported selectively from the blood to the CSF during the early stages of hypoxia.

Therefore, it is suggested that post-mortem CSF PRL levels are markers of hypoxia/ischemia in medico-legal autopsy cases. In addition, the possibility of assessing the severity of hypoxic encephalopathy in the clinical field was also suggested.

## Supporting information

S1 Fig Relationship between the PRL level with collection site, sex, age, survival period, and postmortem period. (TIF)

S2 Fig Immunohistochemical PRL-positivity ratio. (TIF)

S1 Table NCBI reference sequence information of TaqMan assays. (TIF)
