# Supplementary figures and images for: Prolactin selectively transported to cerebrospinal fluid from blood under hypoxic/ischemic conditions
Source: PLoS One. 2018 Jun 27;13(6):e0198673. doi: 10.1371/journal.pone.0198673 (PMC6021042; doi:10.1371/journal.pone.0198673)

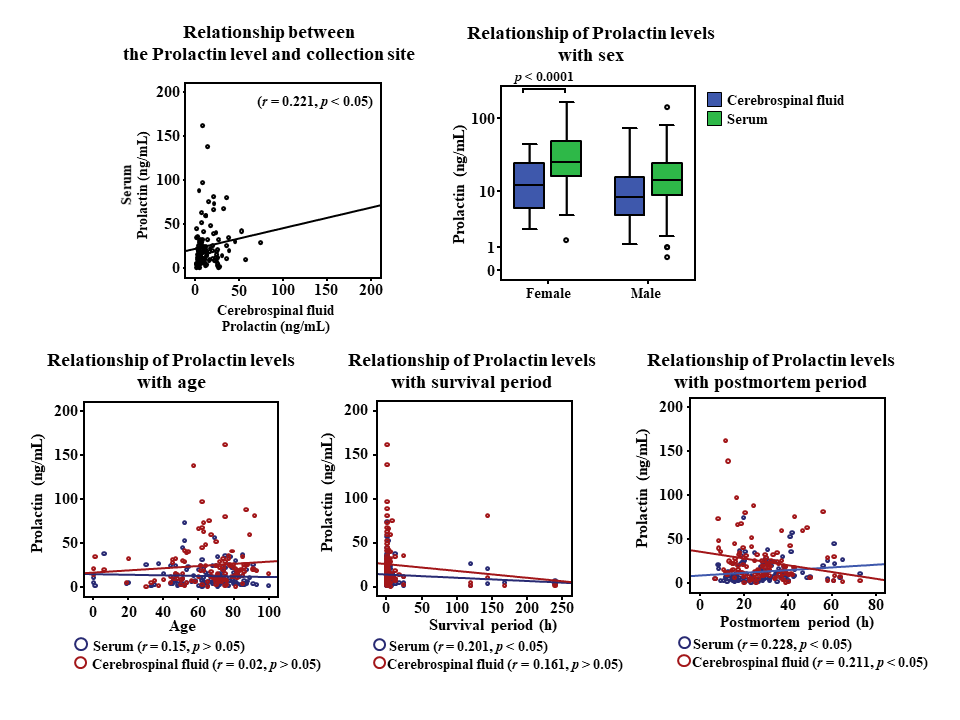

Supplement: S1 Fig — (TIF) [file pone.0198673.s001.TIF]

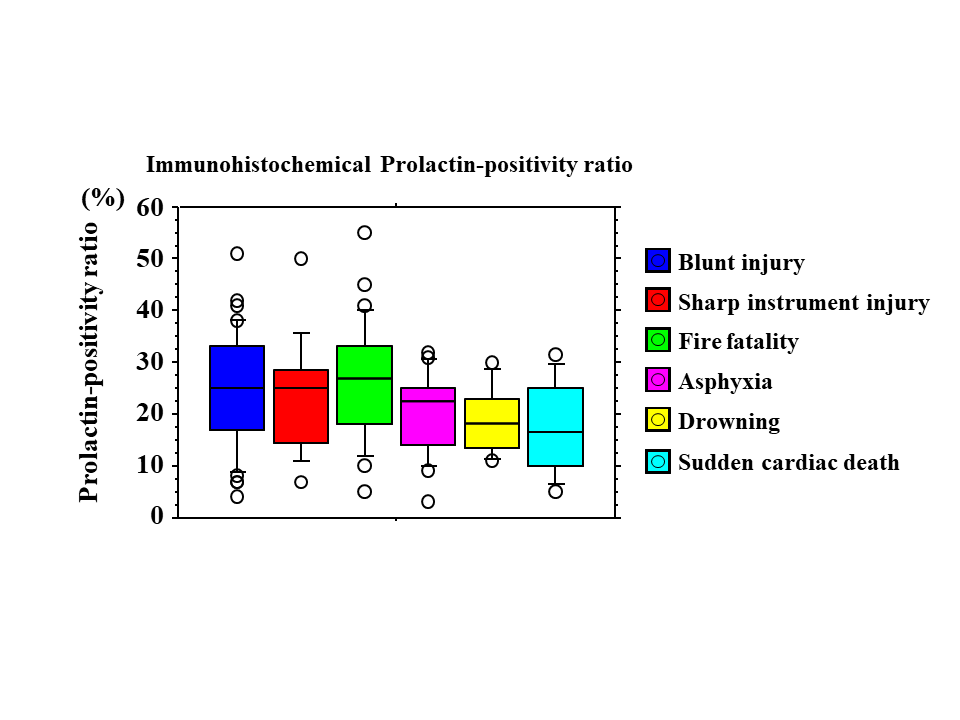

Supplement: S2 Fig — (TIF) [file pone.0198673.s002.TIF]

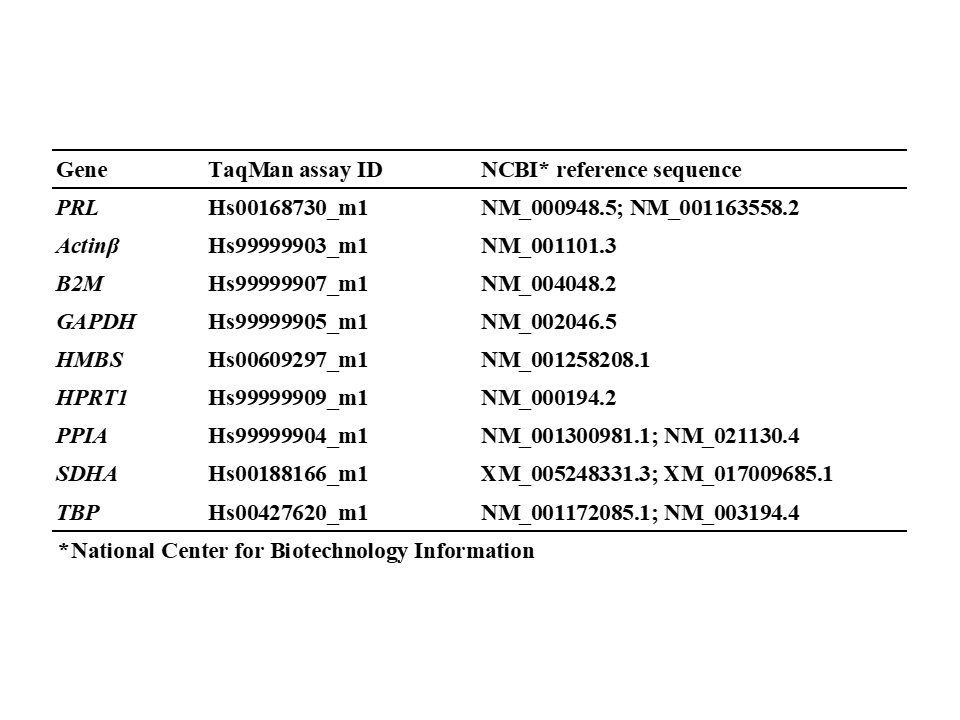

Supplement: S1 Table — (TIF) [file pone.0198673.s003.tif]
